# Improving the vertical solar distiller performance using rotating wick discs and integrated condenser

**DOI:** 10.1007/s11356-022-19873-w

**Published:** 2022-03-31

**Authors:** Mohamed Ragab Diab, Fawzy Shaban Abou-Taleb, Fadl Abdelmonem Essa, Zakaria Mohamed Omara

**Affiliations:** Mechanical Engineering Department, Faculty of Engineering, Kafrelsheikh University, Kafrelsheikh, 33516 Egypt

**Keywords:** Vertical disc solar still, Desalination, Saline water, Rotating parts, Vacuum fan with an external condenser

## Abstract

Freshwater is one of the most essential needs of society. Due to the limited amount of potable water on Earth, guaranteeing the supply of clean water to society is a major challenge. By utilizing abundant sunshine, solar still could be utilized to provide the necessary amount of drinking water in remote locations. The issue of restricted daily production inspires researchers to investigate novel ways for enhancing the thermal performance of desalination techniques while lowering expenses. In this work, the scholars improved a unique distillation method related to solar stills. The authors presented a novel improvement to the vertical distiller design to enhance the exposure area while decreasing the thickness of the water layer as much as possible. Thus, two rotational discs (flat type) covered with wick were integrated into the vertical distiller basin at 1.5 rpm and 5 cm water depth. Furthermore, providing vacuum via a fan with an external condenser. Besides, various rotating speeds (from 400 to 2000 rpm) were tested to determine the perfect fan speed that provides the maximum yield. The experimental findings revealed that the modified vertical distiller produced more pure water than the conventional distiller. Moreover, the rotation of wick discs and vacuum fan enhanced the yield of distillers enormously. Besides, the highest distiller performance was obtained at 1.5 rpm (wick disc speed) and 1600 rpm (fan speed, 10 min ON, and 10 min OFF). Moreover, the daily freshwater output was 19.1 L/m^2^ day for MDSVD without the fan and 23.65 L/m^2^ day for MDSVD with the fan. So, the yield of MSSVD without/with vacuum fan was improved by 548.65% and 660.45%, respectively, over that of CTD. The best thermal efficacy for MDSVD without/with vacuum fan was 77.47% and 84.05%, respectively. Lastly, the average cost of freshwater was 0.021, 0.0177, and 0.0164 $/L for CTD, MDSVD without/with vacuum fan, respectively.

## Introduction

The ability to obtain potable water or drinking water is one of the four major human birthrights (clean air, drinking water, healthy food, and clean energy) (Essa 2022; Saini et al. 2019). The major concern in many nations (both developed and developing) is the scarcity of drinkable water. This issue is becoming more pressing as the world’s population continues to grow, the industrial revolution continues, and agriculture progresses (Essa et al. 2020b). It is predicted that freshwater supplies would be 40% depleted by 2030 (Rijsberman 2006). Desalination of saline, brackish, or even contaminated water is an effective solution to this issue (Panchal et al. 2020). Solar energy is a renewable energy source that may be gathered and used to run thermal desalination systems utilizing simple, inexpensive, and readily accessible equipment (Essa et al. 2020c).

The academics were encouraged to develop novel desalination concepts and technologies to solve the arid region’s potable water dilemma, particularly in Africa (Abdullah et al. 2021a). As a result, scholars proposed different techniques of obtaining drinkable water from saline water as the only way out of the globe’s freshwater crisis. Because it is the most inexpensive, simple, and generally accessible equipment in the thermal desalination group (Bouchekima 2003), solar still was presented as a popular method for water purification (Omara et al. 2021). Nevertheless, solar distillers have a fundamental drawback (Abdullah et al. 2020b; Kabeel et al. 2016): they produce a little amount of drinkable water per day.

In order to improve distiller efficacy and thermal performance, the researchers focus on improving previous designs and implementing new models according to the effective parameters of solar still which include increasing evaporation surface area, studying heat transfer mechanisms, and reducing the depth of water in distillers (Nafey et al. 2000, Tiwari and Tiwari 2006). Consequently, the researchers studied the performance of distiller with fins (Omara et al. 2011), solar still with condensation unit (Kabeel et al. 2014b), distiller with corrugated absorbers (Omara et al. 2015), solar still with reflectors (Omara et al. 2016), distiller with nanoparticles (Kabeel et al. 2014a; Panchal et al. 2021), solar distiller with rotating wick (Abdullah et al. 2019a; Gad et al. 2011; Haddad et al. 2017), tubular solar still (Essa et al. 2021a), drum distiller (Abdullah et al. 2019b, 2021a), trays solar still (Abdullah et al. 2020a; Essa et al. 2021c), disc solar still (Essa et al. 2020d), hemispherical solar still (Attia et al. 2021), pyramid solar still (Alawee et al. 2021a; Essa et al. 2021e), and stepped solar still (Essa et al. 2020a; Gandhi et al. 2021).

The free surface area of evaporation and condensation is one of the most important elements in determining the amount of freshwater produced by the solar still. Many designs and improvements were proposed to improve the solar distillers’ evaporative surface area to enhance yield. Solar distillers with rotating parts (Diab et al. 2021; Essa et al. 2021d) were recommended as a means of achieving this target. The rotating components cause the molecules of water on the surface to break their bonds (surface tension) and decrease the water film as much as possible. Many academics studied the effect of rotational elements on distiller performance such as rotating shaft (Abdel-Rehim and Lasheen 2005, Kumar et al. 2016), fan (Eltawil and Zhengming 2009, Omara et al. 2017), disc (Essa et al. 2020d, 2021d), drum (Abdullah et al. 2019b, 2021a; Alawee et al. 2021b; Essa et al. 2021b; Malaeb et al. 2016), and rotating wick (Abdullah et al. 2019a, 2019b, 2021b; Gad et al. 2011; Haddad et al. 2017; Omara et al. 2021), and these amendments have been more productive than conventional distillers. The surface tension is broken due to the rotation of the elements and the natural mechanism for heat transfer is changed to the forced. These conducts may accelerate the rate of evaporation.

As concluded in a recent review, Diab et al. (2021) discussed the impact of rotating parts inside the different solar distillers. The rotating disc technique was investigated to enhance solar distiller performance. The impact of utilizing a rotating disc inside the basin of a conventional distiller was tested by Essa et al. (2020d). The researchers reported that the disc solar still produced more potable water than the conventional still. Therefore, the corrugated disc distiller with wick was able to produce 124% more distilled water than conventional still at 0.05 rpm. Besides, the corrugated and flat disc distillers covered with wick had an overall efficiency of 54.5% and 50%, respectively. In a recent study, Essa et al. (2021d) investigated experimentally the incorporation of rotating discs inside the vertical distiller at various rotational speeds. The findings indicated that integrating discs within the vertical distiller significantly increased clean water yield as compared to a traditional distiller. Furthermore, the rotary discs’ effective speed was 1.5 rpm at 5 cm saltwater depth which enhanced the yield of modified vertical still by 617.4% over the conventional still.

Moreover, many scholars tested another rotating part (drum) to improve the distiller performance. To enhance the evaporation surface area and reduce the water thickness, Malaeb et al. (2016) proposed a revolving drum inside of the solar distiller. Solar still freshwater production was increased by 250% as a result of this improvement. According to another research by Ayoub et al. (2013), drum speed, basin water depth, and cover cooling are all factors that impact the efficiency of drum solar still. A low drum speed without generating dry spots on the drum’s surface was determined by the authors. In another study, incorporating a rotary fan within the solar still, Kabeel et al. (2012) enhanced potable water output by 25%. Also, Omara et al. (2017) mounted a rotary fan within the solar desalination system and evaluated its effectiveness at different water levels. Scholars indicated that at low fan speeds, the depth of basin water should be as minimal as possible. The drinking water output of the modified distiller combined with a rotating fan was increased by 17%. Furthermore, Essa et al. (2021b) reported observational and analytical research that used a moving drum with two different ends to enhance the productivity of a tubular distiller (closed and opened ends) by 175% at 0.05 rpm with open ends drum and wick.

On another parameter, the capillary effect of wick materials that utilized to cover the moving element made scientifically enhancement the performance of the solar distiller. Haddad et al. (2017) vertically incorporated rotating wick inside the basin of conventional distiller for enhancing the exposed area, evaporating rate, and condensation rate due to water-thin film and wick effect which led to increasing the distiller yield by 14.72% and 51.1% in summer and winter, respectively. In a similar study, installing wick belt on still basin but the belt was horizontal, by Gad et al. (2011), improving the still efficacy up to 43%. Abdullah et al. (2019a, 2021b) tried to maximize the amount of evaporation and exposure area of solar still by utilizing a rotary belt horizontally and vertically at the same time. Controlling the belt speed and its ON–OFF time enhanced the still productivity by 315% at 5 min belt ON and 30 min OFF. Besides, the distillate yield progressed from 4.35 to 7.25 L/m^2^/day via utilizing manual tracking for vertical rotating wick still (Omara et al. 2021) with jute cloth at 0.1 rpm (CCW). Moreover, installing a rotary shaft within the still to increase potable water output by Zeinab and Ashraf (Abdel-Rehim and Lasheen 2005). The best efficacy was enhanced by 5.5% in July.

Considering the impact of vapor withdrawal on still efficacy, Kabeel et al. (2014a) sought to improve the purified water amount through the use of various sorts of nanoparticles with different percentages of the weight fraction. They deduced that the performance was enhanced by 133.64% and 93.87% above traditional still with and without fan by using cuprous oxide–water nanofluid. In another investigation, El-Samadony et al. (2015) investigated experimentally the efficacy of the stepped distiller containing reflector(s) and external condenser. A vacuum fan is attached from the upper back end to the distiller to extract water vapor and transfer it to a condenser. It was shown that a modified distiller with a condenser increased profitability by 165% compared to traditional still if both reflectors and the external condenser were mounted. Moreover, Omara et al. (2015) analyzed the efficacy of the corrugated wick distiller using inner reflectors, combined with external condensers while using various kinds of nanoparticles. Experiments showed that the use of the nanomaterials cuprous and aluminum oxides raises the modified distiller efficiency by around 285.10% and 254.88%, respectively. Also, Kumar et al. (2016) used a stirrer for agitating water in the basin and an external condenser was mounted for withdrawal of some vapor and condensing it.

Regarding the previous concise literature, our recently published review (Diab et al. 2021), and our previous work (Essa et al. 2021d), incorporating rotating discs in vertical distiller augmented extremely the distillate yield of still. However, there was a problem that appeared during experiments on modified double-stage vertical distiller (MDSVD). That problem was the formation of a large amount of vapor in the lower stage, unable to condense due to the hot saltwater in the upper stage. So, the authors installed a vacuum fan with an external condenser for extracting vapor as a step for improving the distiller performance.

Hence, the major aims of this work are to lessen the saltwater layer on rotating discs, capture solar energy as much as possible due to stationary tracking of the sun, eliminate surface tension because of movement, enhance evaporation rate via wick covering discs, and increase the rate of condensation by vapor withdrawal via rotating fan with a condenser. Therefore, the innovation of this study can be summarized as follows:

1. A wick-covered aluminum disc was incorporated into the vertical solar still. A portion of the disc’s lower surface is immersed in saltwater that led to improve the exposed surface of solar radiation and evaporative area in the still basin.
2. The authors evaluated the MDSVD performance compared to conventional tilted distiller (CTD).
3. The impact of utilizing wick was tested at 1.5 rpm for discs and 5 cm water depth.
4. The MDSVD performance incorporated with a rotating vacuum fan with a separate condenser was investigated at numerous fan speeds (400, 800, 1200, 1600, and 2000 rpm) at the same previous conditions for disc and saltwater depth.
5. Controlling the ON–OFF times for rotary fan was tested at two conditions (10 min ON–10 min OFF and 10 min ON–20 min OFF) at the optimum fan speed.

## Design and experimentation of system

### Solar still description

**Fig. 1:**
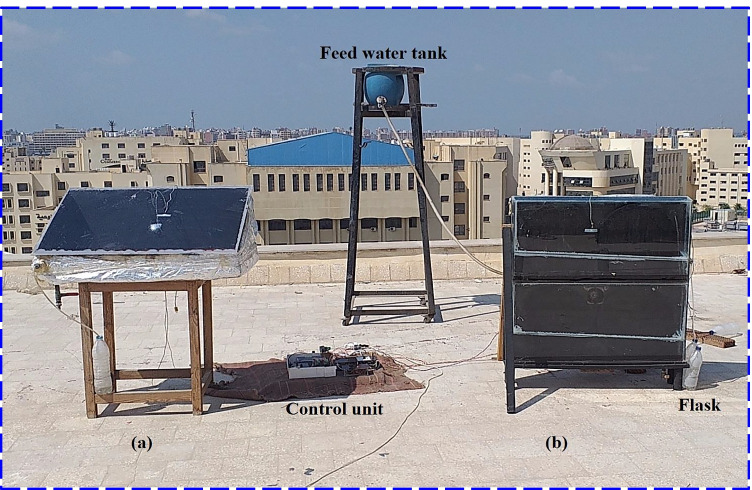
A photograph of the test-rig setup: (**a**) CTD, (**b**) MDSVD

**Fig. 2:**
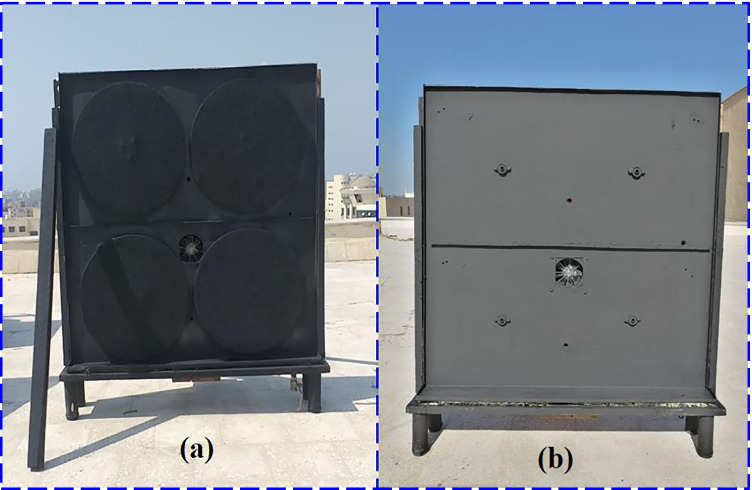
A photo of MDSVD without glass cover: (**a**) with rotating wick discs and vacuum fan, and (**b**) with vacuum fan

As illustrated in Fig. 1 and Fig. 2, the observational tests consisted of two kinds of solar desalination units and a saltwater reservoir feeding both stills. The first distiller was conventional tilted distiller (CTD) that was utilized as a reference for our measurements, and the inclination angle of glass cover was 31°. The second distiller was modified double-stage vertical distiller (MDSVD) that was incorporated with four rotating discs covered with wick and fitted with a vacuum fan. Both distillers were at the same atmospheric conditions to get a fair comparison between their performance.

**Fig. 3:**
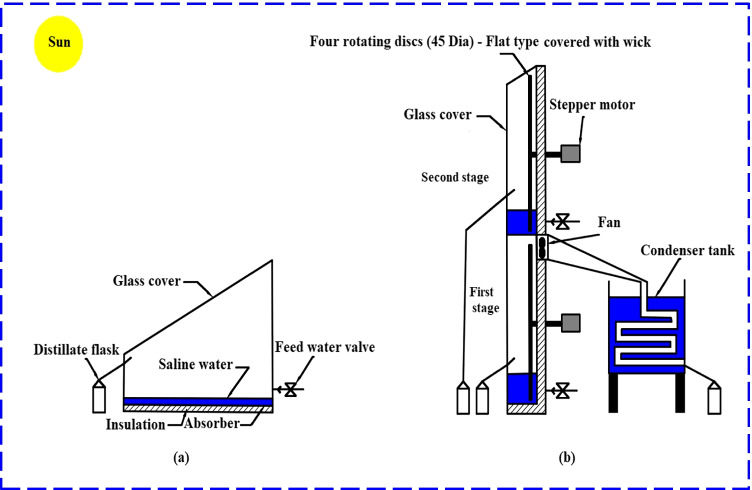
2D layout of test-rig: (**a**) CTD, (**b**) MDSVD

The detailed description of CTD, as seen in Fig. 3, was 1 m long and 0.5 m wide. Besides, the heights were 0.15 m for the lower side and 0.45 m for the upper side. So, the still projected area was 0.5 m^2^. The authors used 1.5-mm galvanized steel for the distiller basin and aimed to capture more solar radiation, so they coated it by matt black paint. Also, the distiller was insulated by 5 cm fiberglass to prevent heat loss from gaps between the still basin and the wooden frame. Subsequently, they covered the solar still by 3-mm glass sheet. Moreover, a sloping trough was used to collect the condensed droplets, which were then sent out of the basin and gathered in graded flasks. On the bottom of the distiller, there was a pipe and a valve that regulated the extra outflow.

**Fig. 4:**
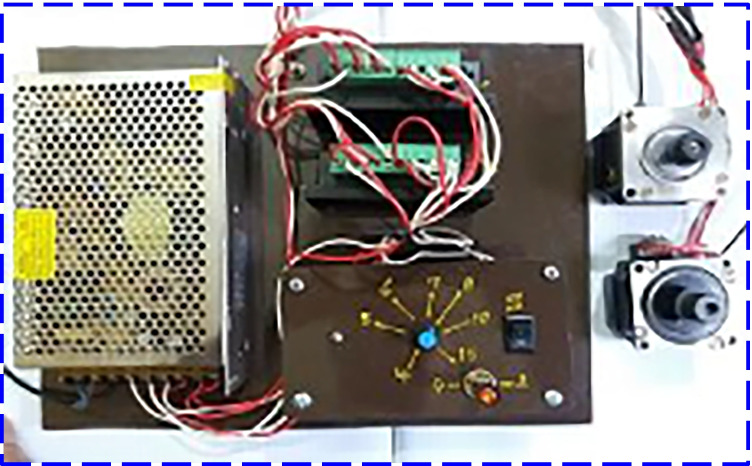
A graph of the control unit of the stepper motor

According to the layout of MDSVD as shown in Fig. 3, the authors reduced the projected area of the modified distiller to 0.1 m^2^. Additionally, they used different types of solar desalination units (vertical distiller). Moreover, the construction of MDSVD was as follows:

1. Double stages of vertical solar still and the dimensions of each stage were 1 m long, 0.1 m wide, and 0.5 high. Besides, the still basin was made of 2-mm galvanized steel and also coated with matt black paint. Furthermore, a 4-mm glass sheet covered the still from four sides as indicated in Fig. 1 for sun tracking. The inclination to the upper base was enough small (tanα = 1/10) to benefit from the condensing water on it. If it was bigger, the amount of vapor would be less due to the reduction in the inner distiller volume and the cover would be very close to the rotating discs. So, the scholars preferred to make this tilt angle of glass cover as tanα = 1/10 to benefit from the condensing water and avoid the demerits of enlarging it as mentioned above. Also, the authors used 4-mm glass sheet for MDSVD to withstand loads (glass weight of four sides and vibration due to rotation). However, the authors used 3-mm glass sheet for CTD still because it has better transparency and the CTD design was simple compared to MDSVD. Finally, the same insulation, feeding, and L-shape wooden frame were used in this distiller.
2. Two rotating discs in each stage were installed on the south side of the still and made of 3-mm black-painted aluminum. The flat discs were 0.45 m in diameter and covered with black jute cotton from all sides. In addition, a stepper motor (6 W—DC) was used to rotate the circular discs via pulley and belt arrangement at 1.5 rpm by the control unit (Fig. 4).
3. The lower stage of MDSVD was integrated with an air suction fan (axial-flow type) that drawled water vapor into the condenser coil which consisted of copper pipe (2 m length and 2 cm diameter) was enclosed in a tank filled with cold water. The condensate water is collected in a graded bottle at the end of the pipe. The blade diameter of the fan was 10 cm, and the authors controlled the fan speed by speed controller from 400 to 2000 rpm (400, 800, 1200, 1600, and 2000 rpm). Besides, the scholars controlled the ON–OFF times for the rotary fan via a timer at two conditions (10 min ON–10 min OFF and 10 min ON–20 min OFF) at the optimum fan speed.

### Experimental tests

Experiments were carried out in the Faculty of Engineering, Kafrelsheikh University, Egypt (latitude = 31.1107° N and longitude = 30.9388° E). The trials were carried out in August and September 2021. All the distillers under consideration were oriented east–west to catch as much of the sun’s energy as feasible. At the same time, factors impacting distiller productivity such as water temperature, ambient temperature, solar radiation, glass temperature, and air speed were measured. In addition, the distilled water production was recorded hourly. Two stills were set on a fair foundation for the operating and environmental standards. The saline water depth within the vertical distiller was 5 cm to cover a larger area of the two rotating discs that are responsible for increasing production in this experiment. The second reason was that the authors utilized 5 cm vertical still to contain the same amount of water as a traditional distiller. The bottom of the discs was immersed in the water basin.

Initially, the bottom disc part was immersed in the disc basin. Hence, the discs started to rotate at the set speed. As a result of movement, the top section of the discs has become a lower portion, and vice versa. Therefore, a thin layer of water formed on the surfaces of the discs. Because it does not take long to warm up, this layer evaporated quickly. In addition, potable water was gathered and recorded daily.

The experimental investigations were performed in some stages. Firstly, the distiller’s performance was examined at constant water depth (5 cm). Besides, flat discs covered with wick were tested at the optimum disc speed (1.5 rpm) from the previous trials. Secondly, flat discs covered with wick and installing a fan with an external condenser were investigated at numerous fan speeds (400, 800, 1200, 1600, and 2000 rpm). Thirdly, the running time of the rotary fan was tested at two conditions (10 ON–10 OFF and 10 ON–20 OFF). Every single modification was examined through an experiment in one complete day.

### Measuring instruments

The instruments used to estimate the factors affecting the output yield of the solar still are K-type thermocouples, van type anemometer, Datalogging solar power meter, and graded flasks. The Datalogging solar power meter was used to measure solar radiation. Temperature analysis at various distiller points was performed by the K-type thermocouples. In addition, the speed of air was measured by a van anemometer. Moreover, a calibrated small bottle was used to record the distillate.

The uncertainty in the measured data was estimated using the method proposed by Holman (2012). The result errors can be estimated by:

1 $${W}_{R}= \sqrt{{\left(\frac{\partial R}{\partial {x}_{1}}{W}_{1}\right)}^{2}+{\left(\frac{\partial R}{\partial {x}_{2}}{W}_{2}\right)}^{2}+\dots +{\left(\frac{\partial R}{\partial {x}_{n}}{W}_{n}\right)}^{2}}$$

where *W*_*R*_ is the resultant uncertainty, and *W*_1_, *W*_2_, *W*_3_, …, *W*_*n*_ are the uncertainties in the independent parameters. The measuring devices’ specifications are listed in Table 1. Furthermore, the hourly yield may be expressed as a function of basin water depth, *m* = *f* (*h*). As a result, the uncertainty for productivity is as follows:

**Table 1:** Values of uncertainty errors and measurement precision for measuring instruments

Measuring devices	Range	Accuracy	Error (%)
Solarimeter K-type thermocouples Anemometer Graded flask	0–5000 W/m^2^ 0–100 °C 0.4–30 m/s 0–2000 mL	± 1 W/m^2^ ± 0.1 °C ± 0.1 m/s ± 1 mL	1.5 1.3 3 2

2 $${W}_{m}= \sqrt{{\left(\frac{\partial m}{\partial h}{W}_{h}\right)}^{2}}$$

Additionally, the uncertainty in thermal efficacy (${\eta }_{th}$) is:

3 $${W}_{{\eta }_{th}}= \sqrt{{\left(\frac{\partial {\eta }_{th}}{\partial m}{W}_{m}\right)}^{2}+{\left(\frac{\partial {\eta }_{th}}{\partial {I}_{R}}{W}_{{I}_{(t)}}\right)}^{2}}$$

As a consequence, the errors in daily productivity and efficacy of distillers are approximately ± 1.4% and ± 2.5%, respectively.

## Results and discussion

Considering various operating scenarios, the implications of rotational wick disc and fan for vapor suction adjustments on the thermal behavior of the still were investigated. Temperature variations, radiation from the sun factors, and daily clean water yield were employed to evaluate the thermal performance of the current still. The thermal efficiency and potable water production output of the current modified and traditional solar stills were analyzed under identical conditions.

### Performance of MDSVD with rotating discs covered with wick at 1.5 rpm

**Fig. 5:**
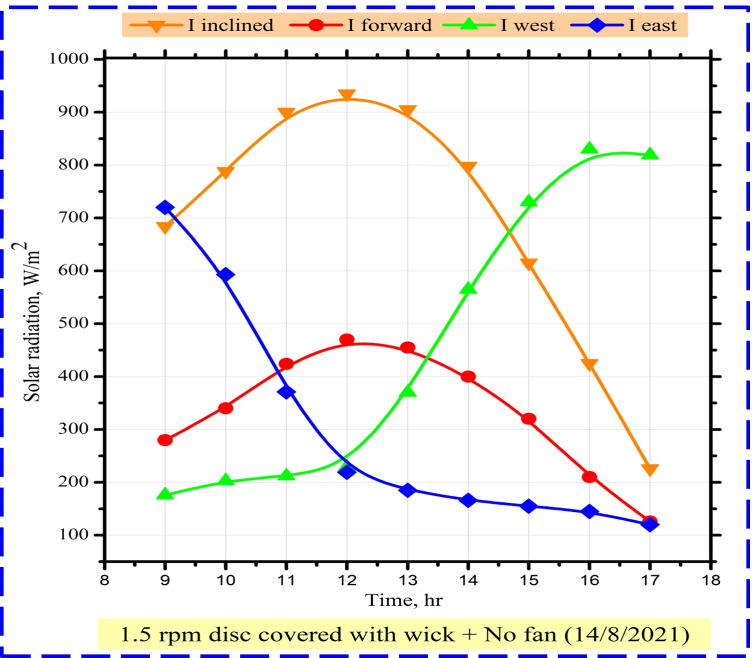
Measured values of solar radiation during 14/08/2021

The authors tested the performance of MDSVD incorporated with rotating wick discs at 1.5 rpm and 5 cm water depth in 14/08/2021 and the measurements for solar radiation and temperature were recorded hourly from 9:00 to 17:00, as shown in Fig. 5. During the measuring period, the sun radiation grew steadily until noon, when it reached its maximum (935 w/m^2^) on the inclined surface. Then, as seen in Fig. 5, its value gradually dropped until it reached its lowest point at sunset. There was a difference between CTD and MDSVD in the amount of solar radiation from one side to another due to the design of the glass cover. Accordingly, solar radiation was measured from inclined, front, east, and west sides (Fig. 5).

**Fig. 6:**
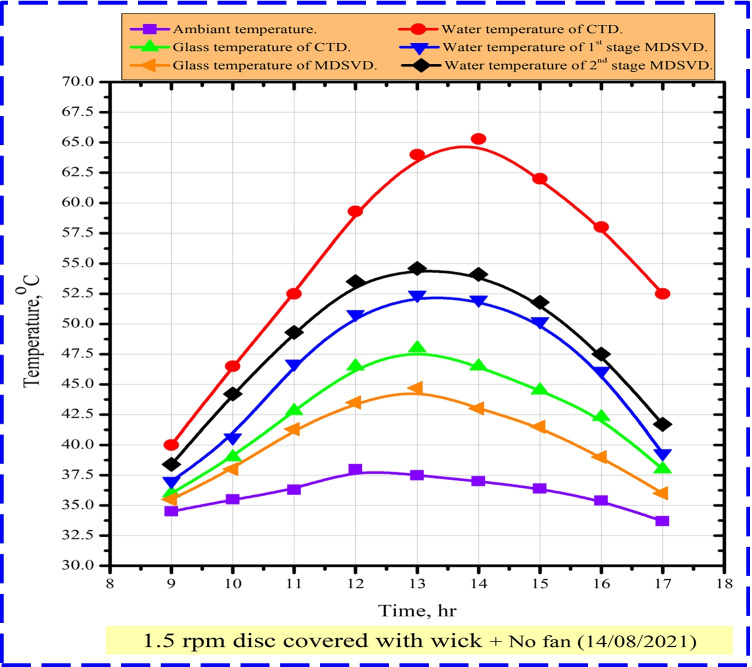
Measuring values of ambient, water basin, and outside glass temperatures

Furthermore, the variations of ambient, water basin, and outside glass cover temperatures were affected by the solar radiation and took the same trend, as illustrated in Fig. 6. The analysis of temperature variations showed that CTD had a higher temperature than MDSVD by about 1.6–13.3 °C due to the angle of receiving direct incident radiation that was responsible for basin water heating. However, MDSVD captured more radiation because of tracking the sun due to its glass design and installing aluminum discs, the wick materials absorbed much solar energy and caused the reduction of water temperatures. Also, the heat loss from MDSVD was higher than CTD because MDSVD was only insulated from the back and bottom sides.

Furthermore, there was a difference between basin water temperatures on the double stages of MDSVD because the water of the upper stage was heated by the vapor of the lower stage due to the glass sheet between them. On the other hand, the glass temperatures of two distillers took the same trend. Besides, CTD glass temperature was higher than that of MDSVD. That is related to basin water temperature and the glass cover area which is exposed to the air. Briefly, the difference between CTD and MDSVD outside glass temperature was about 0.5–3.5 °C.

**Fig. 7:**
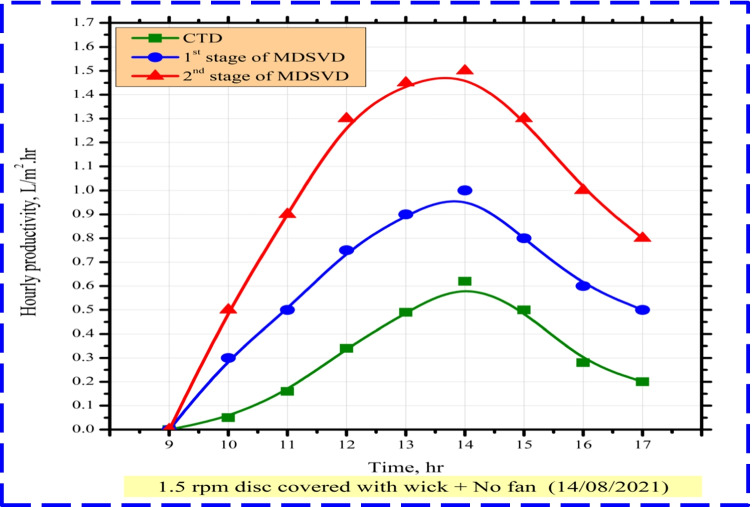
Hourly change of distillate yield for CTD and MDSVD

Comparing hourly distillate yield per 1 m^2^ between CTD and MDSVD with wick, Fig. 7 presents the variation of hourly freshwater output for CTD, lower stage of MDSVD, and upper stage of MDSVD. Nevertheless, as a result of wick discs and saltwater had not yet been heated, there was not enough potable water output in the morning. Henceforth, CTD, 1^st^ stage of MDSVD, and 2^nd^ stage of MDSVD reached their supreme levels of 0.62, 1, and 1.5 L/m^2^ h at 14:00, respectively, as sun radiation increased.

The authors observed that the modifications done on the vertical distiller massively increased the freshwater yield compared to that of CTD. Also, according to solar radiation and temperatures variations (Fig. 5 and Fig. 6), the hourly productivity increased from morning until reached peak as mentioned before. After that, it began to decrease gradually till sunset. Leading reasons for this enhancement include the following: first, the discs played an important role in reducing the water film (main effective parameter of distiller performance) as much as possible. So, the evaporation process happened faster compared to CTD. Also, covering the rotating discs enlarges the wet area of discs taking advantage of the capillary effect property for wick. Second, MDSVD had a huge accessible surface area (0.5 m^2^ for CTD and 1.48 m^2^ for MDSVD, 196% rise) that could capture more radiation from the sun. That led to an enormous rate of evaporation inside MDSVD more than CTD. Third, the surface tension of the water surface was broken due to the movement of discs inside MDSVD basin. Related to turbulence, the water evaporation happened more easily and heightened its rate on MDSVD more than CTD. Forth, the stationary sun tracking of MDSVD increased the absorbed amount of solar radiation (6276 W/m^2^ for CTD, 0.5 m^2^ exposure area vs 16,109 W/m^2^ for MDSVD, 1.48 m^2^ exposure area). Fifth, due to the greater condensation area of MDSVD compared to the conventional still, this milestone was achieved. A condensation process on traditional still happened just on the single-slope glass cover, but on MDSVD occurred on four sides of the glass cover. Besides, the little vibration of the motor helped the condensate droplets to be collected.

**Fig. 8:**
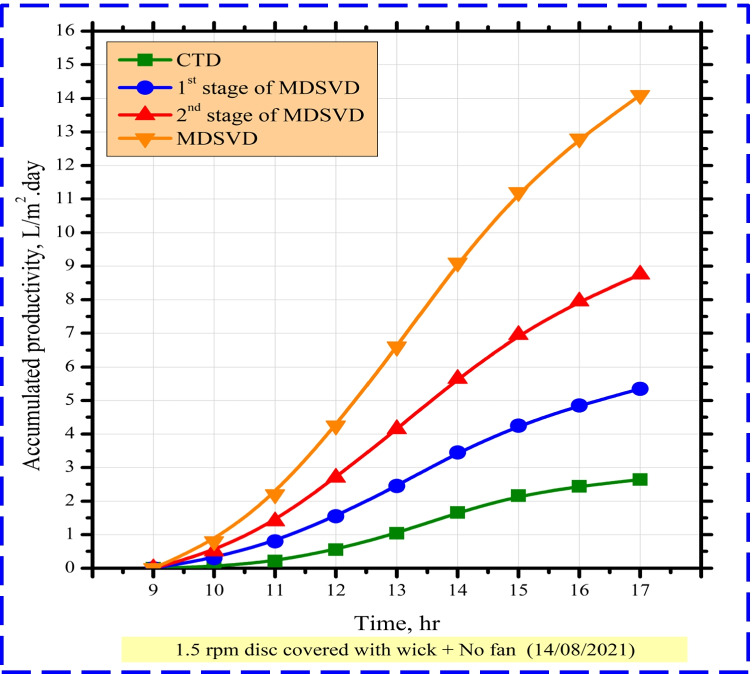
The accumulated distillate output of CTD and MDSVD with wick at 1.5 rpm

Figure 8 presents the hourly accumulated yield for CTD, 1^st^ stage of MDSVD, 2^nd^ stage of MDSVD, and MDSVD. This figure showed that MDSVD had a large clean water output during the whole day compared to CTD. The daily freshwater of CTD, 1^st^ stage of MDSVD, 2^nd^ stage of MDSVD, and MDSVD was 2.96, 7.75, 11.45, and 19.2 L/m^2^ day, respectively. So, the enhancement percentage of MDSVD was 548.65% over CTD output. As discussed in the previous section, the milestones for this high increase could be concluded as follows: minimum water film on discs, the capillary effect of wick, large surface area, large evaporation and condensation rates, movement inside distiller, forced evaporation process, sun tracking, and breaking surface tension.

### Performance of MDSVD with a rotating fan with an external condenser at various fan speeds

The scholars faced a problem that appeared during experiments on modified double-stage vertical distiller (MDSVD). That problem was the formation of a large amount of vapor in the lower stage, unable to condense due to the hot saltwater in the upper stage. So, the authors installed a vacuum fan with an external condenser for extracting vapor as a step for improving the distiller performance. During the investigation, the MDSVD performance with a constant water depth of 5 cm and rotating wick discs at 1.5 rpm was tested to a variety of rotating fan speeds (400, 800, 1200, 1600, and 2000 rpm) to determine the influence of vapor withdrawal on the performance of MDSVD.

According to the trials data, the MDSVD achieved the best performance at the best rotating fan speed (1600 rpm), as shown in Table 2. Additionally, the scholars chose to display the findings at a single speed to highlight the influence of fan adjustment and wick discs on MDSVD performance. Furthermore, Table 2 shows the atmospheric conditions and stills productivity during the testing days at various fan speeds.

**Table 2:** Recorded solar still variables for the several testing days

Day	15/08/2021		400 rpm		1.5 rpm wick disc—latitude = 31.1107° N and longitude = 30.9388° E			
Time	Wind speed, m/s	Ambient temp, °C	Radiation, W/m^2^				Accumulated productivity, L/m^2^ day	
			I inclined	I front	I west	I east	CTD	MDSVD
9:00	1.2	31.5	680	280	165	710	0	0
10:00	0.9	32.5	814	340	185	580	0.06	0.8
11:00	1.7	34.9	905	410	200	335	0.26	2.45
12:00	5.5	35	930	435	212	215	0.62	4.7
13:00	2.7	36	907	425	312	200	1.22	7.6
14:00	1.9	35.4	830	375	557	195	1.8	10.3
15:00	2.4	35.3	650	305	720	180	2.3	12.7
16:00	4.4	35	430	200	820	153	2.53	14.3
17:00	2.2	33.7	235	125	809	120	2.69	15.55
Day	24/08/2021		800 rpm		1.5 rpm wick disc—latitude = 31.1107° N and longitude = 30.9388° E			
Time	Wind speed, m/s	Ambient temp, °C	Radiation, W/m^2^				Accumulated productivity, L/m^2^ day	
			I inclined	I front	I west	I east	CTD	MDSVD
9:00	2.5	33.5	681	280	176	720	0	0
10:00	1.2	34.4	795	340	203	593	0.06	0.75
11:00	2.8	34.7	905	424	212	371	0.19	2.25
12:00	1.9	35	940	470	229	219	0.51	4.5
13:00	2.3	35.5	895	455	370	185	0.95	7.1
14:00	1.4	34.5	790	400	565	166	1.52	9.85
15:00	2.3	33.7	610	320	730	155	2.04	12.3
16:00	3.3	33.4	420	210	830	145	2.41	14.3
17:00	2.5	33	230	126	819	120	2.59	15.8
Day	28/08/2021		1200 rpm		1.5 rpm wick disc—latitude = 31.1107° N and longitude = 30.9388° E			
Time	Wind speed, m/s	Ambient temp, °C	Radiation, W/m^2^				Accumulated productivity, L/m^2^ day	
			I inclined	I front	I west	I east	CTD	MDSVD
9:00	2.2	33.5	690	280	165	725	0	0
10:00	2.5	34.2	815	394	177	600	0.05	0.8
11:00	3.9	35	927	512	186	407	0.22	2.4
12:00	2.5	36.5	950	570	230	220	0.54	4.75
13:00	1.4	38	920	550	395	200	1.08	7.6
14:00	1.9	37	830	490	565	190	1.68	10.85
15:00	2.7	36	675	407	723	175	2.18	13.7
16:00	3.3	34	449	270	825	153	2.54	15.95
17:00	2.5	33	230	140	820	120	2.73	17.6
Day	29/08/2021		1600 rpm		1.5 rpm wick disc—latitude = 31.1107° N and longitude = 30.9388° E			
Time	Wind speed, m/s	Ambient temp, °C	Radiation, W/m^2^				Accumulated productivity, L/m^2^ day	
			I inclined	I front	I west	I east	CTD	MDSVD
9:00	0.9	33.3	690	345	165	715	0	0
10:00	1.5	34.6	785	400	180	565	0.06	0.8
11:00	0.3	35	890	495	191	405	0.25	2.65
12:00	0.7	36.4	945	536	230	215	0.65	5.4
13:00	2.4	37	905	529	385	200	1.2	8.5
14:00	0.5	36.2	791	485	605	191	1.8	11.8
15:00	2.2	36.1	608	385	730	172	2.27	14.6
16:00	0.8	34.8	430	229	820	142	2.61	16.65
17:00	2.5	33	230	140	760	120	2.78	18.3
Day	30/08/2021		2000 rpm		1.5 rpm wick disc—latitude = 31.1107° N and longitude = 30.9388° E			
Time	Wind speed, m/s	Ambient temp, °C	Radiation, W/m^2^				Accumulated productivity, L/m^2^ day	
			I inclined	I front	I west	I east	CTD	MDSVD
9:00	1	32	690	345	165	715	0	0
10:00	1.5	34.6	825	435	180	565	0.06	0.8
11:00	1.4	35	990	560	201	410	0.26	2.65
12:00	2.8	35.5	1003	585	257	224	0.72	5.3
13:00	4.3	34.8	955	548	414	212	1.36	8.4
14:00	5.7	33.3	868	500	615	200	1.94	11.35
15:00	2.7	33	658	405	755	170	2.38	14.05
16:00	3.3	32.9	449	270	825	153	2.68	16.05
17:00	2	32	230	140	820	120	2.82	17.65

As discussed in the previous section, there was another reason for the increase of MDSVD output. The non-condensable vapor from the lower stage of MDSVD is exhausted to the exterior condensation unit through the small power fan. Furthermore, the fan drawled non-condensable vapor from the basin and transported it to the condensation unit. The impact of non-condensable vapor, which reduced condensation ratio, is also eliminated. In addition, the fan circulated air within the MDSVD lower stage. Consequently, the evaporation rate was enhanced due to the air circulation and vapor withdrawal caused by the fan. Because of this difference in evaporation and condensation capabilities, MDSVD produced at a quicker and higher rate than the traditional one.

**Fig. 9:**
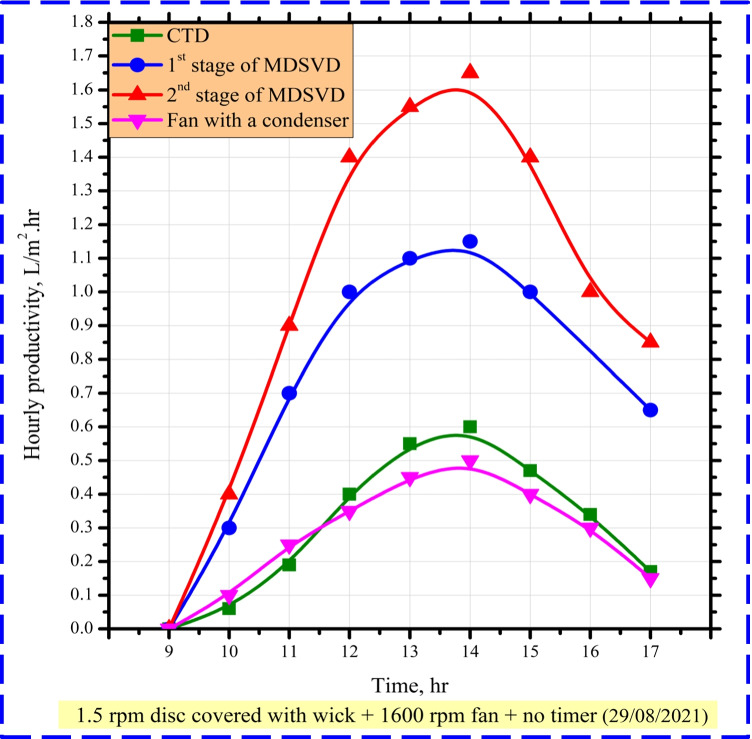
The impact of continuous running fan on the hourly output of MDSVD related to CTD

**Fig. 10:**
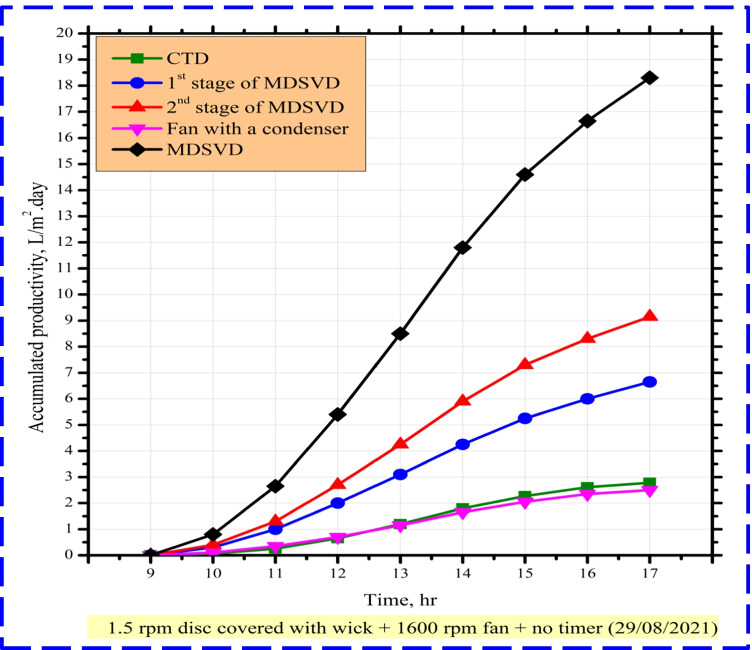
The impact of continuous running fan on accumulated output of MDSVD related to CTD

The impact of installing a fan was shown obviously on the hourly and accumulated productivity of MDSVD, as indicated in Fig. 9 and Fig. 10, respectively.

Comparing hourly distillate yield per 1 m^2^ between CTD and MDSVD with wick and continuous rotating fan with a condenser, Fig. 9 presents the variation of hourly freshwater output for CTD, lower stage of MDSVD, upper stage of MDSVD, and rotating fan. Nevertheless, as a result of wick discs, negative pressure due to the fan and saltwater had not yet been heated, there was not enough potable water output in the morning. Henceforth, CTD, 1^st^ stage of MDSVD, 2^nd^ stage of MDSVD, and rotating fan reached their supreme levels of 0.6, 1.15, 1.65, and 0.5 L/m^2^ h at 14:00, respectively, as sun radiation increased.

Figure 10 presents the hourly accumulated yield for CTD, 1^st^ stage of MDSVD, 2^nd^ stage of MDSVD, continuous rotating fan with a condenser, and MDSVD. This figure showed that MDSVD had a large clean water output during the whole day compared to CTD. The daily freshwater of CTD, 1^st^ stage of MDSVD, 2^nd^ stage of MDSVD, continuous rotating fan with a condenser, and MDSVD was 3.08, 8.95, 11.25, 2.9, and 23.1 L/m^2^ day, respectively. So, the enhancement percentage of MDSVD was 650% over CTD output and the consumed power of the fan was 44Wh. As discussed in the previous sections, the milestones for this high increase could be concluded as follows: minimum water film on discs, the capillary effect of wick, large surface area, large evaporation and condensation rates, movement inside distiller, forced evaporation process, sun tracking, vapor withdrawal, and breaking surface tension.

### Performance of MDSVD at the best fan speed and controlling fan ON–OFF times

In order to increase the distiller efficacy, the authors aimed to control the ON–OFF times to decrease the consumed power of rotating fan. So, the study investigated the best fan speed (1600 rpm) at various conditions (continuous fan speed at 1600 rpm, 1600 rpm at 10 ON–10 OFF times, and 1600 rpm at 10 ON–20 OFF times), as presented in Table 3.

**Table 3:** Documented solar still parameters for the various testing days

Day	29/08/2021		1600 rpm – continuous		1.5 rpm wick disc—latitude = 31.1107° N and longitude = 30.9388° E			
Time	Wind speed, m/s	Ambient temp, °C	Radiation, W/m^2^				Accumulated productivity, L/m^2^ day	
			I inclined	I front	I west	I east	CTD	MDSVD
9:00	0.9	33.3	690	345	165	715	0	0
10:00	1.5	34.6	785	400	180	565	0.06	0.8
11:00	0.3	35	890	495	191	405	0.25	2.65
12:00	0.7	36.4	945	536	230	215	0.65	5.4
13:00	2.4	37	905	529	385	200	1.2	8.5
14:00	0.5	36.2	791	485	605	191	1.8	11.8
15:00	2.2	36.1	608	385	730	172	2.27	14.6
16:00	0.8	34.8	430	229	820	142	2.61	16.65
17:00	2.5	33	230	140	760	120	2.78	18.3
Day	01/09/2021		1600 rpm + 10 ON–10 OFF		1.5 rpm wick disc—latitude = 31.1107° N and longitude = 30.9388° E			
Time	Wind speed, m/s	Ambient temp, °C	Radiation, W/m^2^				Accumulated productivity, L/m^2^ day	
			I inclined	I front	I west	I east	CTD	MDSVD
9:00	0.7	33.6	684	282	175	716	0	0
10:00	1.3	34.1	788	352	204	590	0.06	0.85
11:00	0.9	35.6	905	425	213	372	0.23	2.95
12:00	0.7	36.4	968	476	230	220	0.56	5.6
13:00	0.6	37	918	455	370	200	1.03	8.7
14:00	1.4	35.8	815	422	615	185	1.65	11.9
15:00	1.2	34.1	658	362	755	170	2.23	14.8
16:00	0.9	32.8	449	268	820	155	2.63	17
17:00	2.5	32	230	140	803	119	2.81	18.8
Day	31/08/2021		1600 rpm + 10 ON–20 OFF		1.5 rpm wick disc—latitude = 31.1107° N and longitude = 30.9388° E			
Time	Wind speed, m/s	Ambient temp, °C	Radiation, W/m^2^				Accumulated productivity, L/m^2^ day	
			I inclined	I front	I west	I east	CTD	MDSVD
9:00	0.9	32	680	340	160	700	0	0
10:00	1.5	33.8	825	435	175	565	0.05	0.8
11:00	1.3	34.7	910	500	190	350	0.22	3
12:00	0.9	35	980	555	201	208	0.56	5.5
13:00	4.7	34.1	957	550	345	195	1.04	8.6
14:00	2.6	32.3	817	485	575	190	1.66	11.75
15:00	1.8	32	650	401	755	167	2.2	14.3
16:00	3.5	31.7	425	272	811	140	2.6	16.45
17:00	2.5	31	230	135	800	115	2.79	18.1

In the next sections, the best condition will be declared obviously by figures to avoid reputations.

### Daily productivity rise

**Fig. 11:**
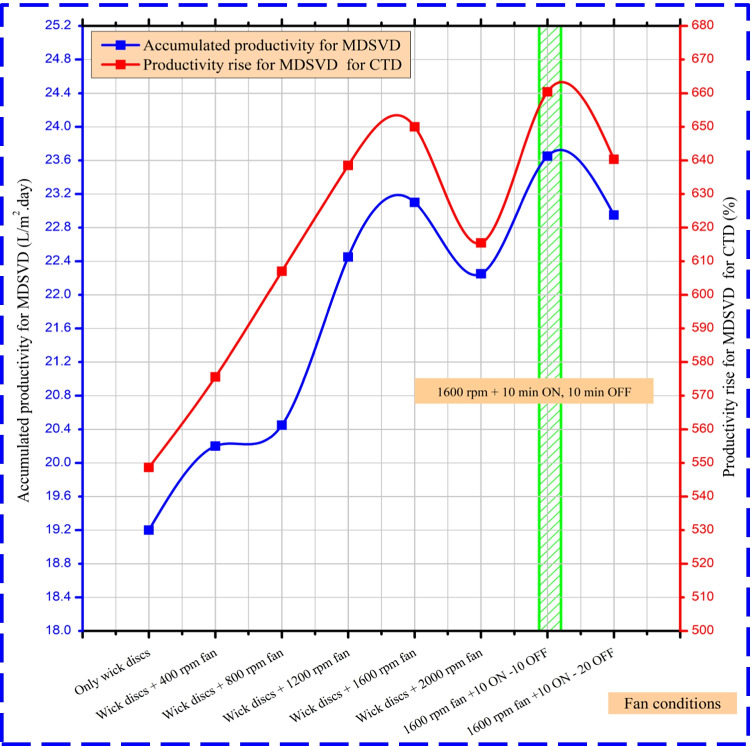
The impact of various fan conditions on MDSVD daily productivity and enhancement ratio

The rise in daily productivity for MDSVD at several speeds of the rotating fan and ON–OFF conditions is shown in Fig. 11. The daily improvement in productivity, as indicated in the figure, is dependent on the control parameters of the rotation speed of the fan, and also the adjustment performed on the vertical distiller such as rotating discs at 1.5 rpm and wick effect. The output of clean water will increase when the fan speed is increased from 400 rpm to the maximum productivity of 1600 rpm. Then, when the speed changes from 1600 to 2000 rpm, it begins to drop. Besides, the controlling of fan ON–OFF times improved the distillate yield of MDSVD. The authors compared the productivity of MDSVD to that of CTD under the same conditions in each experimental test to ensure a fair comparison even if the weather changed from one day to the next because it would change by the same value on both distillers.

The maximum daily productivity rise was obtained for MDSVD over CTD at 1.5 rpm rotating wick discs, as shown in Fig. 11. The greatest yield was gained at 1600 rpm fan running 10 ON–10 OFF times, where the output gain for MDSVD was approximately 660.45% (23.65 L/m^2^ day) compared to that of CTD (3.11 L/m^2^ day). Moreover, the freshwater was reduced at higher fan speeds compared to 1600 rpm as the quicker rotation of the fan provided high vapor flowrate and depleted more thermal energy from the distiller.

### Daily thermal efficiency

The daily efficacy, $\upeta$_**d**_, of the solar still is calculated from the following equation (Essa et al. 2020d):

4 $${\upeta }_{\mathbf{d}}=\frac{\sum \dot{\mathrm{m}}(\mathrm{daily\;yield }) \times {\mathrm{h}}_{\mathrm{fg}}(\mathrm{vaporization\;latent\;heat})}{\mathrm{\Sigma A}(\mathrm{projected\;area\;of\;still}) \times \mathrm{ I}\left(\mathrm{t}\right)(\mathrm{solar\;intensity})+\mathrm{\;Motor\;energy}}$$

**Fig. 12:**
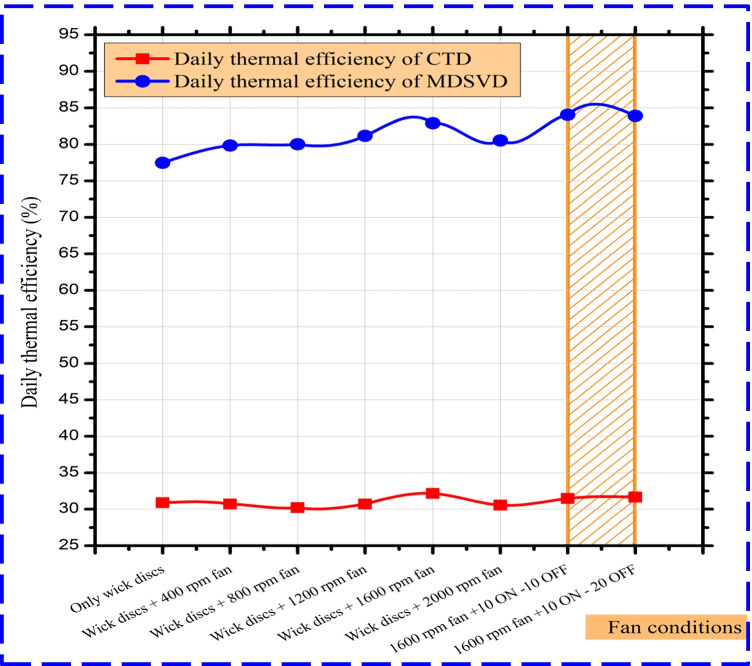
The impact of rotating fan on MDSVD daily efficacy vs CTD

In this section, the daily efficacy of the investigated solar stills in the various investigated scenarios is explained in Fig. 12. The scholars observed that the efficacy of CTD varied from 30.89 to 32.15% during tests. Moreover, the MDSVD efficacy recorded its peak value (84.05%) at the same conditions following Fig. 10.

### Relation of the current research to prior studies

Prior studies are compared to the present study’s results to identify how much growth can be made with the redesigned vertical distiller. As shown in Table 4, our results were compared to those of the prior publications. Using a traditional solar still as a reference, the efficacy of the incorporated modifications was assessed by comparing.

**Table 4:** Comparison of the present and prior investigations’ outcomes

Classification	Authors	Enhancement	Efficacy	Cost
Drum distiller	Abdullah et al. (2019b)	350%	85.5%	0.039 $/L
	Malaeb et al. (2016)	250%	-	-
	Abdullah et al. (2021a)	296%	79%	0.041 $/L
	Alawee et al. (2021b)	214%	65%	-
Rotating disc distiller	Essa et al. (2020d)	124%	54.5%	-
Vertical distiller with rotating discs	Essa et al. (2021d)	617.4%	77.2%	0.019 $/L
Solar distiller with moving wick	Haddad et al. (2017)	14.72% and 51.1% in summer and winter, respectively	65%	0.011 $/L
	Gad et al. (2011)	-	43%	-
	Abdullah et al. (2021b)	300%	82%	0.031 $/L
	Omara et al. (2021)	7.25 L/m^2^ day	51%	0.02 $/L
	Abdullah et al. (2019a)	315%	84%	0.032 $/L
Solar distiller with a rotating shaft	Abdel-Rehim and Lasheen (2005)	0.47 and 0.52 L/m^2^ day in July for the rotary shaft and packed layer, respectively	-	-
	Kumar et al. (2016)	39.49%	30.57%	10 Rs/L (= ~ 0.13 $/L)
Solar distiller with vibratory harmonic effect	Eldalil (2009b; 2009a; 2010)	132%	60%	-
Solar distiller with water fan driven by a wind turbine	Kabeel et al. (2012)	25%	38%	0.0447 $/L
	Omara et al. (2017)	17%	39.8%	-
	Eltawil and Zhengming (2009)	29.17% and 32.93% without and with sun tracking	62.01% and 62.38% without and with sun tracking	0.0743 $/L
The current study	Vertical solar still with rotating discs covered with wick and a fan with condenser	660.45%	84.05%	0.0164 $/L

### Evaluation of 1 L cost of distillate water

The economic study for the distillers under consideration mainly focuses on the distiller classification including its elements, presented in Table 5.

**Table 5:** Manufacture cost of 1 m^2^ distillers

Unit	CTD ($)	MDSVD ($)
Iron sheet Aluminum disc Glass sheet Ducts and support legs Paint insulation (Fiberglass) Production Wick and supports DC-Motor and connections Fan and external condenser Total capital cost (F)	30 - 10 25 10 7 20 - - - 102	165 40 70 45 30 35 50 10 80 75 600

In order to approximate the cost of freshwater provided by CTD and MDSVD, the analytical formulas have been demonstrated as seen below (Abdullah et al. 2020b):

5 $$\mathrm{The\;capital\;recovery\;factor\;}\left(\mathrm{CRF}\right)=\frac{{\mathrm{i}(1+\mathrm{i})}^{\mathrm{N}} }{{(1+\mathrm{i}) }^{\mathrm{N}}-1}$$

where *N* is the still lifetime (number of years), and (*i*) is the interest rate.

6 $$\mathrm{The\;fixed\;annual\;cost }\left(\mathrm{FAC}\right)=\mathrm{F}*(\mathrm{CRF})$$

where *F* is the distiller capital cost ($).

7 $$\mathrm{The\;sinking\;fund\;factor\;}\left(\mathrm{SFF}\right)=\frac{\mathrm{i }}{{(1+\mathrm{i}) }^{\mathrm{N}}-1}$$

8 $$\mathrm{The\;salvage\;value\;}\left(\mathrm{S}\right)=0.2*\mathrm{F}$$

9 $$\mathrm{The\;annual\;salvage\;value }\left(\mathrm{ASV}\right)=\mathrm{S}*(\mathrm{SFF})$$

10 $$\mathrm{The\;annual\;maintenance\;and\;operating\;costs\;}\left(\mathrm{AMC}\right)=0.15*(\mathrm{FAC})$$

11 $$\mathrm{The\;total\;annual\;cost\;}\left(\mathrm{TAC}\right)=\mathrm{FAC}+\mathrm{AMC}-\mathrm{ASV}$$

12 $$\mathrm{The\;freshwater\;cost }\left(\mathrm{CPL}\right)\mathrm{\;in\;\$}/\mathrm{L}=\mathrm{TAC}/\mathrm{M}$$

where *M* is the average freshwater yield in year.

The considerations of the cost analysis are as follows: the interest rate is 15%, the number of working days per year is 340 days (sunny days in Egypt), and the distiller lifetime is 10 years. The daily productivity is approximately 3.11, 19.2, and 23.65 L/m^2^ day for CTD and MDSVD without/with fan, respectively. As a result, the cost of freshwater produced by CTD and MDSVD without/with fan was estimated to be 0.021, 0.0177, and 0.0164 $/L, respectively.

## Conclusion

The performance of the vertical distiller incorporated with rotating discs covered with wick and a rotary fan with an external condenser was experimentally investigated and compared with a conventional still. The main parameter tested in this study was the rotational speed variations of fan. Mainly focused on the above-mentioned discussed findings, it is declared that:

1. The integration of rotating fan within the lower stage of MDSVD increased dramatically the yield of purified water and thermal energy efficacy due to minimum water film on discs, the capillary effect of wick, large surface area, large evaporation and condensation rates, movement inside distiller, forced evaporation process, sun tracking, vapor withdrawal, and breaking surface tension.
2. The daily accumulated freshwater output was 3.11, 19.2, and 23.65 L/m^2^ day for CTD and MDSVD without/with fan, respectively. In addition, for the modified distiller, the highest amount of yield was gained at 1600 rpm for fan and 10 ON–10 OFF times. As a result, the freshwater distillate of MDSVD without/with fan was improved by 548.65% and 660.45% compared to that of CTD, respectively.
3. The supreme thermal efficacy for CTD and MDSVD without/with fan was 31.04%, 77.47%, and 84.05%, respectively.
4. The costs of freshwater obtained from CTD and MDSVD without/with fan are found to be 0.021, 0.0177, and 0.0164 $/L, respectively.

### *Scope for further research*

There are a variety of reasons why solar stills with rotating components should be studied in greater depth:

- To improve the absorption and evaporation areas, different rotating disc geometries are indeed being investigated.
- The solar still may be fitted with a solar tracking device to ensure that it receives consistent energy from the sun.
- As a heat storage material in the still, sensible materials and nanoparticles can be utilized to store energy.

## Data Availability

Not applicable.

## English symbols

A: Projected area of still (m^2^)
AMC: The annual maintenance and operating costs ($)
ASV: The annual salvage value ($)
CPL: The freshwater cost ($/L)
CRF: The capital recovery factor
F: The distiller capital cost ($)
FAC: The fixed annual cost ($)
h_fg_: Latent heat of vaporization (KJ/kg)
i: The interest rate (%)
I(t): Solar intensity (W/m^2^)
$$\dot{\mathrm{m}}$$: Hourly distillate yield (mL/m^2^ h)
M: The average freshwater yield in year (L/m^2^ year)
N: The still lifetime (years)
S: The salvage value ($)
SFF: The sinking fund factor
TAC: The total annual cost ($)

## Greek symbols

Η: Efficiency

## Abbreviations

CTD: Conventional tilted distiller
MDSVD: Modified double-stage vertical distiller
